# A community-based cluster randomised controlled trial to evaluate the effectiveness of different bundles of nutrition-specific interventions in improving mean length-for-age z score among children at 24 months of age in rural Bangladesh: study protocol

**DOI:** 10.1186/s12889-017-4281-0

**Published:** 2017-05-02

**Authors:** Sk Masum Billah, Tarana E. Ferdous, Mohd Anisul Karim, Michael J. Dibley, Shahreen Raihana, Md Moinuddin, Nuzhat Choudhury, Tahmeed Ahmed, D. M. Emdadul Hoque, Purnima Menon, Shams El Arifeen

**Affiliations:** 1 Maternal and Child Health Division, icddr,b, 68 Shahid Tajuddin Ahmed Sarani, Mohakhali, Dhaka, 1212 Bangladesh; 2 Nuffield Department of Population Health, University of Oxford, Oxford, UK; 3 Sydney School of Public Health, Sydney Medical School, the University of Sydney, Sydney, Australia; 4 Department of Statistical Science, University of Padova, Padova, Italy; 5 Nutrition and Clinical Services Division, icddr,b, 68 Shahid Tajuddin Ahmed Sarani, Mohakhali, Dhaka, Bangladesh; 6 International Food Policy Research Institute (IFPRI), Washington, DC USA

**Keywords:** Randomised controlled trial, Bundling, Nutrition interventions, Stunting, Length-for-age, First 1000 days of life

## Abstract

**Background:**

Prevalence of stunting among under-five children in Bangladesh is 36%, varying with geographic and socio-economic characteristics. Previously, research groups statistically modelled the effect of 10 individual nutrition-specific interventions targeting the critical first 1000 days of life from conception, on lives saved and costs incurred in countries with the highest burden of stunted children. However, primary research on the combined effects of these interventions is limited. Our study directly addresses this gap by examining the effect of combinations of 5 *preventive* interventions on length-for-age z-scores (LAZ) among 2-years old children.

**Methods:**

This community-based cluster randomised trial (c-RCT) compares 4 intervention combinations against one comparison arm. Intervention combinations are: 1) Behaviour change communication (BCC) on maternal nutrition during pregnancy, exclusive breastfeeding, and complementary feeding, along with prenatal nutritional supplement (PNS) and complementary food supplement (CFS); 2) BCC with PNS; 3) BCC with CFS; and 4) BCC alone. The comparison arm receives only routine health and nutrition services. From a rural district, 125 clusters were selected and randomly assigned to any one of the five study arms by block randomisation. A bespoke automated tab-based system was developed linking data collection, intervention delivery and project supervision. Total sample size is 1500 pregnant women, with minimum 1050 resultant children expected to be retained, powered to detect a difference of at least 0.4 in the mean LAZ score of children at 24 months, the main outcome variable, between the comparison arm and each intervention arm. Length and other anthropometric measurements, nutritional intake and other relevant data on mother and children are being collected during enrolment, twice during pregnancy, postpartum monthly till 6 months, and every third month thereafter till 24 months.

**Discussion:**

This c-RCT explores the effectiveness of bundles of *preventive* nutrition intervention approaches addressing the critical window of opportunity to mitigate childhood stunting. The results will provide robust evidence as to which bundle(s) can have significant effect on linear growth of children. Our study also will have policy-level implications for prioritising intervention(s) tackling stunting.

**Trial registration:**

The study was retrospectively registered on May 2, 2016 and is available online at ClinicalTrials.gov (ID: NCT02768181).

**Electronic supplementary material:**

The online version of this article (doi:10.1186/s12889-017-4281-0) contains supplementary material, which is available to authorized users.

## Background

Globally in 2013, 161 million children under the age of five years (U-5 children) had a height-for-age Z score more than 2 standard deviations (SD) below the median of WHO Multicentre Growth Reference Study (MGRS) Child Growth Standard (termed ‘stunting’), and half of these children resided in Asia [1]. Although the stunting prevalence is decreasing worldwide, reductions in South Asia have been slower (36% since 1990) compared to 57% reduction in Europe and more than 70% in East Asia and the Pacific [2]. Stunted children have higher mortality [3], lower academic attainments [4], short stature as adults [5], reduced economic productivity [6], reduced cognition [7] and have a higher risk of cardio-metabolic diseases in adulthood [8], all contributing to the ‘stunting syndrome’ [9], and the trans-generational cycle of undernutrition and poverty [10]. Thus, stunting is a major public health problem in South Asia. In Bangladesh, the latest Demographic and Health Survey (2014) [11] reports that stunting prevalence has decreased to 36.1%. Despite a reduction in the past decade [12], national stunting prevalence remains high [13], with higher levels in rural areas (37.9%) and among the poorest populations (50.2%). Trends also show widespread geographic disparity - stunting has declined by about 2–3 percentage point per year in most regions except no change in *Sylhet* division, the north-eastern region [11].

### Global goals related to stunting

Although unaddressed by the Millennium Development Goals, reduction of stunting prevalence became the focus of several high-profile nutrition initiatives such as Scaling Up Nutrition (SUN, 2010) [14], World Health Assembly 2012 [15] and Nutrition for Growth 2013 [16]. The World Health Assembly 2012 has a target of 40% reduction of U-5 stunting by 2025. Hence, the recent Sustainable Development Goals (SDGs) adopted by the UN in 2015 included stunting reduction as one of their goals (SDG 2.2) [17].

### The critical window of opportunity

Childhood stunting is considered to have in utero origins [18]. The first 1000 days of life (conception to postpartum two years) is regarded as a critical ‘window of opportunity’ for growth and development [19]. The long-term interactions of both proximal (i.e. biological and environmental) and distal (i.e. socioeconomic) factors play significant roles during this period in influencing the linear growth of children [20]. The proximal factors of childhood stunting in South Asia include undernourished mothers, limited dietary diversity during pregnancy, poor infant and young child feeding practices (IYCF) including suboptimal breastfeeding and/or complementary feeding, and inappropriate sanitation and hygiene [21]. However, even short-term improvements in nutritional status (in utero and postnatal) can result in substantial mean height gain of the child even within a single generation [22].

### Potential interventions to tackle stunting

In the Lancet Maternal and Child Nutrition Series 2013, Bhutta et al. (2013) statistically modelled the effect of 10 nutrition-specific interventions on lives saved and economic costs in 34 countries containing the majority of the children with stunted growth [23]. They suggested that at an estimated additional cost of $9.6 billion per annum for 90% coverage of these interventions, one-fifth of stunting among the U-5 children can be averted. However, there is a need for primary research to test and clarify the combined impact of nutrition-specific interventions as packages. They agreed about this gap and that further trials are needed.

### Relevance of the present study

We have adapted five *preventive* interventions to create our own interventions bundles, based on Bhutta et al.’s list considering feasibility of scale-up in low resource settings and targeting the first 1000 days of life (from conception to two years of age). Our interventions include, a) Behaviour change communication (BCC) on nutrition and health-related practices during pregnancy; b) BCC on exclusive breastfeeding (EBF) for postnatal first 6 months; c) BCC on age-specific complementary feeding (CF) with continued breastfeeding thereafter till 23 completed months; d) Nutritional supplementation during pregnancy (PNS) with preventive doses of micronutrients, and partial provision of protein and lipids; e) Nutritional supplementation for children (CFS) during 6 to 23 completed months of age, with preventive doses of micronutrients, and partial provision of protein and lipids. In essence, this randomized controlled trial provides a crucible to see how different nutritional interventions interact when delivered in bundled packages in community settings to affect linear growth of the child from conception to two years of age compared to the comparison arm where routines practices will run unhindered.

### Study aim and hypothesis

This study aims to test the effectiveness of the intervention bundle(s) on improving length-for-age Z score (LAZ) of children at two years of age. We hypothesised that our intervention bundle(s) would cause a change of at least 0.4 in mean LAZ of children, translating to at least 30% reduction in stunting in that arm(s), compared to that in the comparison arm.

## Methods

### Study design

**Fig. 1:**
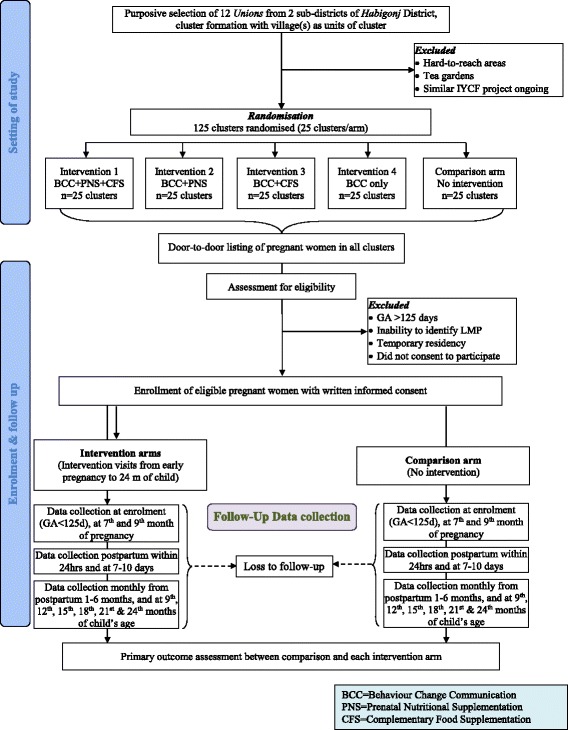
CONSORT flow diagram of the study

This cRCT evaluates the effectiveness of 4 different combinations of the selected pre- and post-natal nutrition-specific interventions for improving children’s linear growth, compared to the comparison arm with usual practices. The three types of BCC are combined into one ‘BCC’ intervention and delivered universally in all four intervention arms. Profile of the four intervention arms are – intervention arm 1: BCC with PNS and CFS; intervention arm 2: BCC with PNS; intervention arm 3: BCC with CFS; intervention arm 4: BCC alone. (Fig. 1 **)**. The cohort of the mother-child dyads will be followed-up over the intervention period of ~36 months starting from recruitment to 24 months of child’s age.

### Study setting

The study is being conducted in the *Habiganj* district of *Sylhet* division in Bangladesh where stunting prevalence has remained stagnant over a decade [11]. Further, the prevalence of household food insecurity was highest (77%) and the prevalence of household food deficit was second highest (33%) in Sylhet [24].

### Cluster randomization and intervention allocation

**Fig. 2:**
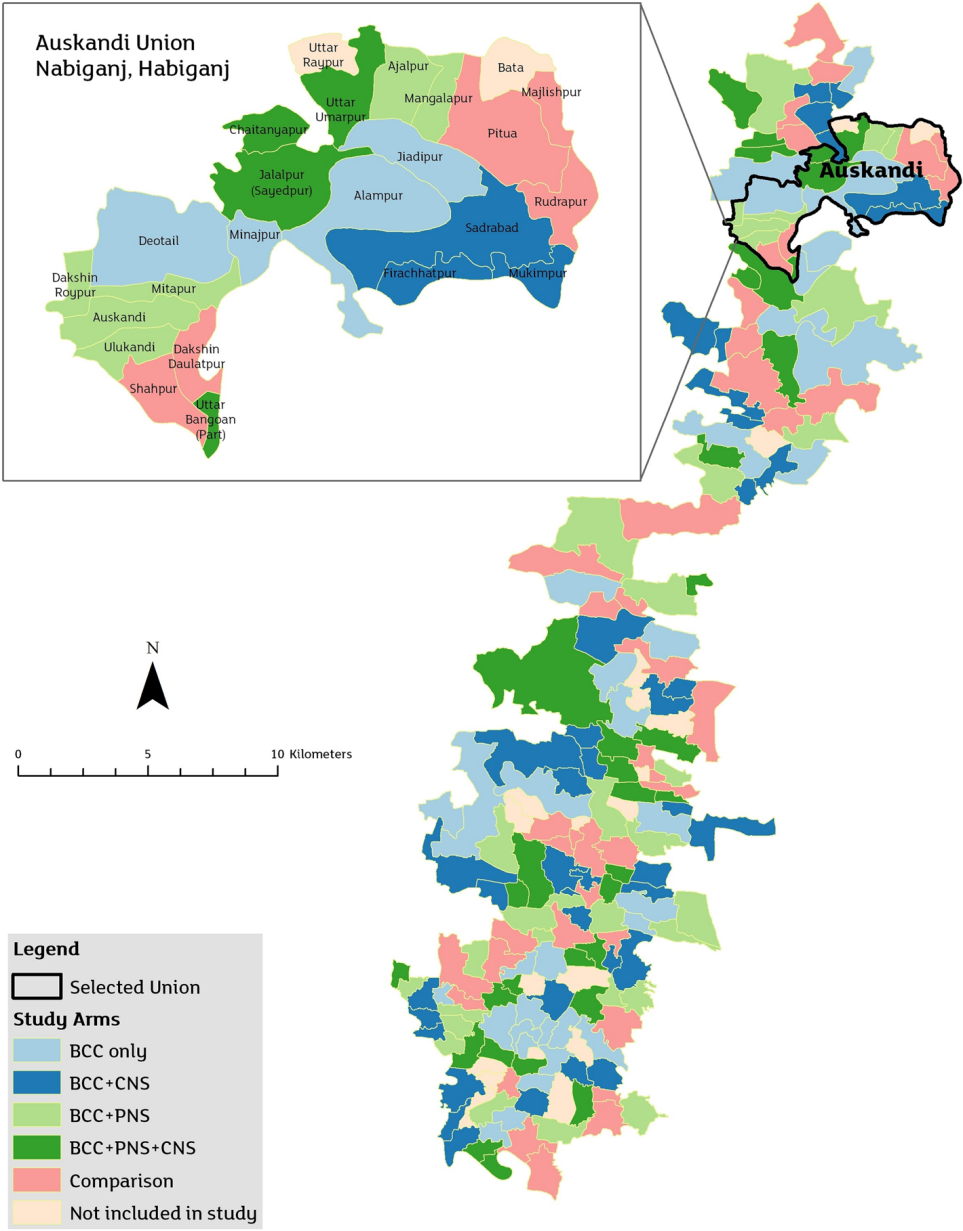
Study clusters in five arms with close-up view of one union for illustration

We purposively selected two adjacent sub-districts (*Bahubal* and *Nabiganj*) of *Habiganj* district. These two sub-districts have a total of 20 unions (lowest administrative unit). We excluded 8 unions, 3 being in hard-to-reach areas (seasonal flooding and poor road transport system) and 5 having intensive nutrition intervention projects implemented by other non-government organisations (NGOs). The remaining 12 unions had enough population to cover the desired sample size. Each union was then divided into clusters, each cluster comprising of ~450 households or ~2000 population (average 2–3 villages). We selected five clusters or multiples of 5 clusters from each union, 125 clusters in total following the cluster exclusion criteria detailed in Table 1. We then applied block randomisation by generating random sequence of 1–5 (each number representing a specific study arm) of 25 blocks with the analytical software Stata SE 14.2. Clusters were then allocated to arms, five at a time, based on the random sequence (Fig. 2). Taking equal numbers of clusters per arm from each union will ensure neutralizing the effect of variations, if any, of background characteristics between the unions. Allocation concealment was impractical due to community-based study design and nature of BCC intervention. However, care is taken to keep the outcome assessors blinded to the interventions allocated to specific clusters. The intervention and the evaluation teams have minimal contact to minimise information bias.

**Table 1:** Inclusion and exclusion criteria

Exclusion criteria for clusters	
• Similar nutrition interventions currently being implemented by either government or non-government agencies in the selected cluster • The cluster is too hard to reach • The cluster includes tea gardens. Communities in tea gardens comprise of unique ethnicity, culture and lifestyle for which our intervention is not customized.	
Inclusion criteria for participants	
• All newly identified pregnant women aged 15 to 49 years • Gestational age ≤ 125 days • Permanent residents of the study area.	
Exclusion criteria for participants	
• Woman could not recall last menstrual period (LMP). • Woman not a permanent resident of the study area

### Sample size

Each study cluster comprised of an average population of 2000 and necessary adjustments were considered for cluster randomization. Crude birth rate (CBR) in rural Bangladesh in 2011 was 23 live births per 1000 population [25]. Therefore, taking six months of recruitment window for pregnant women, each cluster would have yielded ~23 new pregnancies on average.

Several assumptions were considered for the sample size calculation [26]. The first assumption was that mean LAZ (our primary outcome variable) for 18–23 months old children in the comparison arm will be −2.0, and standard deviation of mean LAZ in both intervention and comparison arms will be 1, based on the mean HAZ reported in this age group in the BDHS 2011. These BDHS 2011 [25] parameters were the latest available during this protocol development. The estimates were still valid in 2015 since the latest BDHS (2014) showed similar stunting rates in *Sylhet* division [11]. Second, a 0.4 difference in mean LAZ score was expected in intervention arms compared to comparison. As the third assumption, we considered an 80% power and 5% alpha; the inter-cluster correlation coefficient of 0.06, derived from the design effect for HAZ in BDHS 2011, was used for adjustment of clustering effects. Fourth, the ratio between 4 intervention and 1 comparison arms was set at 1:1:1:1:1. Taking all the above assumptions into account, the Stata SE 13 software calculated the sample size to be 175 children per arm, which can be yielded from 25 clusters. However, to examine the secondary outcome variables between the comparison arm and individual intervention arm with a higher statistical power, the sample size in the comparison arm was doubled, thus the ratio between participant numbers in each intervention arm and comparison arm was raised to 1:2, respectively. The final allocation ratio of study participants was thus 1:1:1:1:2 for intervention arm 1: intervention arm 2: intervention arm 3: intervention arm 4: comparison arm 5. Thus, the final sample size was 175 children in each intervention arm and 350 children in the comparison arm. Considering the characteristics of the intervention, however, enrolment was during pregnancy. We therefore considered the following losses- 11% pregnancy loss due to abortion and still births (personal communication from Health and Demographic Surveillance System run by icddr,b at Mirzapur, Tangail), a 4% mortality in the first two years of life [25], 15% losses due to non-consent to participate by eligible women and loss-to-follow up. Loss-to-follow up includes refusal and migration out of study area. Thus, final sample size considering participant recruitment during pregnancy was 250 pregnant women in each intervention arm and 500 pregnant women in the comparison arm which is expected to yield the required number of children at 24 months from the enrolled cohort.

### Participant recruitment

We enrolled 10 pregnant women in each intervention cluster and 20 in each comparison cluster, following the inclusion and exclusion criteria for participant eligibility (Table 1). Data collectors systematically conducted door-to-door surveys of all households in the study area to identify newly pregnant women, which were repeated in subsequent monthly cycles. The woman’s pregnancy status was assessed by enquiring about the first date of her last menstrual period (LMP), and possible pregnancy was confirmed with a sensitive pregnancy urine test kit (Excel®). Women tested positive were invited to voluntarily participate in the study with appropriate informed written consent. Recruitment of eligible pregnant women started in November 2015 and was completed in May 2016. Follow up of the cohort is ongoing.

### Intervention plan

#### Intervention packages - behaviour change communication (BCC) through home-based counselling

The nutrition-specific BCC modules are adapted from the WHO-UNICEF published ‘Key messages booklet on the community infant and young child feeding counselling package’ [27] with linguistic help from the nationally adapted and validated BCC modules [28, 29]. The modules combine the three BCC types: i) Maternal nutrition during pregnancy; ii) Infant nutrition through exclusive breastfeeding; iii) Child nutrition through age-appropriate complementary feeding during 6–23 completed months. During pregnancy, counselling sessions include BCC on nutrition during pregnancy; and sensitization for WHO-recommended optimal breastfeeding practices. Starting immediately after birth of offspring to postnatal 5th month, intensive counselling and hands-on demonstrations are conducted every month of optimal breastfeeding practices (including positioning and attachment, expression and storage of breast milk), and nutrition of lactating mothers. From 6th month, counselling and hands-on demonstration sessions are conducted every three months and include preparation and feeding of optimal, age-appropriate and nutritious complementary food made from local ingredients, responsive feeding, continued breastfeeding, and nutrition of lactating mothers. Additional counselling visits are made if requested by respondents or if CHW deems the respondent is resistant to change their current practices. As integral parts of nutrition, counselling on good hygiene and care-seeking practices for both mother and child are also delivered in each session.

#### Intervention packages - nutritional supplementation

The prenatal and complementary food supplements being provided were previously tested in a supplement trial in Bangladesh [30], and are provided in selected clusters randomised to the three arms to receive either one or both supplements (Fig. 1).

#### Nutritional supplements for pregnant women

A lipid-based nutritional supplement (LNS) developed by Nutriset™, packed in ~20 g sachets, is provided to pregnant women. The formulation contains vegetable fat (soy), skimmed milk powder, peanuts, vitamin and mineral complex, sugar, stabilizer: fully hydrogenated vegetable fat, and antioxidant: tocopherols. An additional file details the components (see Additional file 1). Each sachet contains 70–75% of the recommended dietary allowance (RDA) for most of the micronutrients, and partial provision of protein and lipids, including essential fatty acids. The dose is one sachet per pregnant woman per day throughout pregnancy.

#### Complementary food supplements for children

Another LNS formulation developed by Nutriset™, packed in each ~10 g sachets, is provided to be fed to children from 6 to 23 completed months of age. The formulation contains vegetable fat, skimmed milk powder, peanuts, sugar, vitamin and mineral complex, maltodextrin, and emulsifier: lecithin. An additional file details the components (see Additional file 2). Each 20 g of LNS contains 70–75% of RDA for most of the micronutrients, and partial provision of protein and lipids, including essential fatty acids. The dose is two sachets per child per day, starting from 6 completed months of age (180 days) continued till 23 completed months.

### Comparison arm (natural practice)

In the comparison arm, study participants are receiving usual nutrition interventions available from routine health services only. Pregnant women are eligible to receive nutrition counselling on maternal and child nutrition during antenatal check-up (ANC) visits and caregivers of children under two years of age receives IYCF counselling during postnatal care visits within 42 days and sick child management visits at health facilities and by NGO workers (if available) [31]. However, contact coverage and content quality of these platforms and interventions is very low and does not reach those who do not seek the services. Antenatal iron-folic acid supplements are available during ANC contacts at public health facilities. Children aged 6–59 months receive vitamin A supplements during biannual national vitamin A campaigns, although coverage is unsatisfactory [11].

### Field implementation of interventions

Counselling and supplement sachets are delivered through our specially trained Community Health Workers (CHWs). Their tasks are supervised and monitored by two layers of field supervisors – a team of Field Supervisors (FSs), and a Field Research Assistant (FRA). Minimum educational qualification of the CHWs is Higher Secondary School Certificate (HSC) completed, while the FSs and the FRA have completed their Bachelor’s degree. The intervention team was given intense counselling training in three phases, and monthly meetings are held for field-related troubleshooting and as refresher courses. Supervisors provide daily feedback to associated CHWs during on-site counselling supervision.

At least 4 antenatal counselling sessions are delivered during pregnancy. Following live birth notification, 8 counselling sessions are delivered within 48 h and 7–14 days of birth, and at completion of every month till 6 months. Thereafter, counselling is delivered every third month till postnatal 23 completed months (Table 1). The CHWs also distribute supplement sachets every month, and provide counselling on the importance and instructions on supplement taking and storage.

### Evaluation plan

A range of quantitative data collection methods, including structured questionnaire and anthropometric measurements, have been adopted for assessments of primary and secondary outcomes. A timeline depicts the schedule of data collection in Table 2.

**Table 2:**
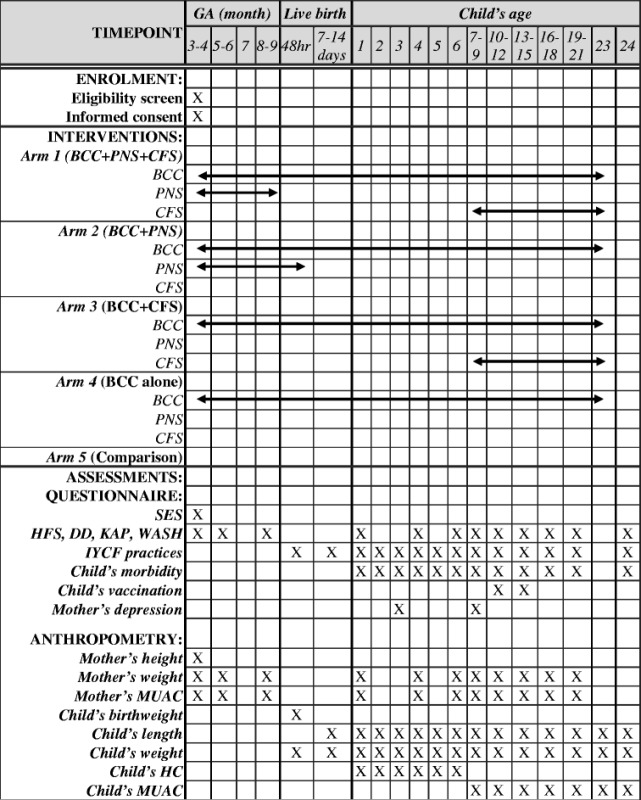
Schedule of enrolment, interventions, and assessments of the study

*GA* Gestational age, *BCC* Behaviour Change Communication on Nutrition and health, *PNS* Prenatal Nutritional Supplementation, *CFS* Complementary Food Supplementation, *SES* Socio-economic status, *HFS* Household food security, *DD* Dietary diversity, *KAP* Knowledge, attitude and practice on nutrition, *WASH* Water, sanitation and hygiene, *IYCF* Infant and young child feeding, *MUAC* Mid upper arm circumference, *HC* Head circumference

### Trial outcomes and measurements

#### Primary outcomes

The primary outcome measure will be the LAZ of children at 24 months of age based on WHO MGRS 2006 child growth reference. This will be calculated from the child’s age in months and length on associated ages. Length is measured at age 7–10 days, then monthly from 1 to 6 months, and at 9^th^, 12^th^, 15^th^, 18^th^, 21^st^ and 24^th^ months of age, with locally manufactured collapsible length boards (precision 1 mm).

### Secondary outcomes

#### Gestational weight gain of mothers

Body weight of mothers is measured during enrolment and on the 6th and 9th month of gestation. Gain in weight will be compared among homogenous gestational ages and initial nutritional status of mothers. Weight is measured with a Tanita™ weighing scale (precision 100 g).

#### Weight of children

Body weight of children is measured at birth, on 7–10 days, then monthly from 1 to 6 months, and every third month thereafter till 24 month, using Salter scales (precision 10 g).

##### Nutritional intake of mothers

Detailed ingredients of the pregnant mothers’ dietary intake on the previous 24-h are collected, through an interviewer-administered open 24-h dietary recall form adapted from the FAO dietary diversity guidelines [32], during enrolment and on the 6th and 9th month of gestation. Further, maternal food intake is collected every third month after birth till 24 months. Ingredients will be collated into food groups to calculate dietary diversity score.

#### Infant and Young Child Feeding (IYCF) practices

Data on IYCF practices (breastfeeding and complementary feeding) are collected through a structured IYCF questionnaire, employing 24-h [11] and 7 day recall methods. WHO-recommended core indicators of optimal breastfeeding include early initiation of breastfeeding (within 1 h of birth), EBF till 6 months after birth and continued breastfeeding till at least 2 years post-birth [33]. Information on all these indicators and associated information are collected. Data on early initiation of breastfeeding and pre-lacteal use is collected within 24-h of birth and on the 7th day. EBF information is collected periodically on the 7th day of birth, and then monthly postpartum from 1 to 6 months. Continued breastfeeding information is collected every three months thereafter till 24 months.

WHO-recommended complementary feeding practices include introduction of solid, semi-solid or soft foods; minimum dietary diversity, meal frequency and acceptable diet; and consumption of iron-rich or iron-fortified foods from postnatal 6 completed months [33]. This information is collected every third month from postnatal 6 to 24 months. Comparison of continued breastfeeding and complementary feeding practices will be made between intervention arms and the comparison arm at these time points.

#### Other measurements

Background characteristics of study participants: Information on demographic and socio-economic status (SES), family members, reproductive and morbidity history of pregnant women were collected using a structured questionnaire [25]. Household food security (HFS) information was collected through a questionnaire adapted from food security and nutrition surveillance tool [34]. These were recorded once during recruitment of the pregnant women after consent has been taken, to compare background characteristics between the arms.

Water sanitation and hygiene (WASH) and Knowledge attitude and practice (KAP): A questionnaire on the core indicators on water, sanitation and hygiene (WASH) was developed based on a standard manual for WASH [35]. Woman’s knowledge, attitude and practices (KAP) regarding maternal and child nutrition was developed based on WHO-recommended IYCF indicators and KAP manual [36]. WASH and KAP are collected during pregnancy and postpartum follow up visits.

Childhood morbidity: Morbidity, especially diarrhoea, directly impedes growth, while better hygiene practices lower infectious disease burden [37]. Therefore, children’s morbidity data is collected every month from postnatal 1 to 6 months and every three months thereafter till 24 months, using a 2-week recall questionnaire [11]. Vaccination data would be collected at 12 and 15 months based on EPI schedule.

Additional anthropometric measurements: Mother’s height is measured during recruitment for body mass index (BMI) calculation during early pregnancy. Mother’s mid-upper arm circumference (MUAC) (precision 1 mm), infant’s head circumference (during 1–6 moths age) and infant’s MUAC (during 7–24 months) are collected during follow-up visits, for associated z-score calculations.

### Data collection

All questionnaire-based data and anthropometric measurements are collected by trained data collectors. Anthropometric measurements are taken following measurement-specific standard operating procedure [38]. Measuring tools are calibrated regularly. All data collectors are female and locally recruited. Before initiation of participant recruitment, the data collection team was intensely trained by the central team on all aspects of data collection, including consent taking, different data collection methods, anthropometric measurements, question throwing techniques, use of the tablet and cultural aspects to consider during data collection. Monthly meetings are held for troubleshooting and refresher trainings are conducted as needed. Following the initial training, all data collectors were standardised for anthropometric measurements against two gold standard trainers from icddr,b. Subsequently, refreshers trainings are conducted every two months to retain measurement standards. The data collectors are directly supervised by two FRAs who monitor data collection quality and provide on-site feedback as needed. One field manager is responsible for overall field coordination.

### Project management system for implementation and monitoring

A bespoke automated Project Management Information System (MIS) has been developed linking data collection and intervention delivery through android-based tablet (handheld)-PC with a central database where progress can be monitored by the field supervisors and central staff. On pregnancy enrolment through the tab, an android platform-based application automatically generates subsequent prenatal visit plans and tools at pre-specified schedules and tasks of CHWs and data collectors (intervention modules for CHWs and questionnaire for data collectors). Similarly, birth is registered in the tab immediately upon notification. To ensure post-partum data collection within 24 h of delivery, a mobile phone-based birth-notification system has been established. Enrolled pregnant women and their family members are continuously encouraged to notify birth by text message or voice calls as early as possible, preferably within six hours of birth. Registration of live births then generates appropriate plans and tools for postpartum visits by CHWs and data collectors. The web-linked desktop dashboard system is used to monitor activities/performance of CHWs and data collectors in real time by field supervisors and the central team.

### Process evaluation

#### Adherence to supplements for both mothers and children

Empty supplement sachets are collected and supplement tracking is carried out in each visit by the CHWs. Besides, we are assessing supplement compliance using a structured questionnaire with some open questions with queries on respondents’ attitude and practice towards the antenatal and complementary food supplements provided to them. We would collect similar data for checking compliance of complementary supplements to children aged 6 months to 24 months (in associated arms).

#### BCC monitoring

The BCC modules set in the tablets are customised for each CHW. Date and time of each visit is automatically recorded as the CHW proceeds with the module. The module for each visit has several sub-topics linked to associated details, and timing of these sub-topics is also automatically recorded. These measures ensure an automated machine-based monitoring system from which BCC delivery coverage can easily be tracked and calculated.

### Statistical analysis plan

Data analysis to detect change of the primary outcome (mean LAZ) will be conducted with length measured at 1, 3, 6, 9, 12, 15, 18, 21 and 24 months of age using t-tests assuming LAZ will satisfy the normality assumption. Longitudinal assessment of significance using lag time of stunting (mean length > −2SD below median of WHO reference population) will be done by the log rank test, followed by fitting a Cox proportional hazards model for each intervention arm to determine the change in risk of stunting compared to the comparison arm. Multiple linear regression will be employed to quantify the effect of intervention in each intervention arm compared to the comparison arm in terms of mean LAZ change and some secondary continuous outcome measures including changes in mother’s weight, birth weight, diarrhoea morbidity. Logistic regression will be used on binary outcome variables which comprise most of our secondary outcomes measures (e.g. – EBF and breastfeeding initiation within 1 h of birth). All these models will be controlled for possible confounders (household food security, educational background and SES) if randomisation of the clusters does not control for differences in these factors at baseline. For regression models, we shall consider random effects of clustering. All analysis will be intention-to-treat. Quantitative data analysis will be done using Stata SE 14.2 (StataCorp LP, College Station, Texas, USA).

## Discussion

Our primary study aims to investigate the effects of 5 nutrition-specific *preventive* interventions, adapted from the Lancet’s modelled intervention list (Bhutta et al. [2013]), delivered in different bundles to improve the LAZ score from conception to two years of age in a food-insecure area of rural Bangladesh.

One of the major strength of this study is its depth (multiple community-friendly evidence-based intervention packages) and breadth (first 1000 days with interventions directed at both mother and child). The results will attempt to clarify the effects of counselling combined with prenatal nutritional and/or complementary food supplementation on linear growth of the child. In addition, our study will also explore the interventions impact on nutritional intake during pregnancy, gestational weight gain, birth weight and IYCF practices. The cluster RCT design in a community setting will offer robust evidence on the effectiveness of the bundles compared to the comparison arm where routine practices will run unabated. The relatively homogenous study population in a food-insecure area would further strengthen our inferences, as we would expect minimal inter-cluster differences that would otherwise potentially confound associations.

We are also one of the few studies in Bangladesh using a bespoke digital platform for intervention delivery and monitoring of coverage. Android-based handheld devices are being used by CHW to automate planning of visit schedules as well as aid counselling sessions by providing the appropriate BCC content based on the gestational age and subsequently, age of the child. Visual aids and video demonstrations of good practices included in the electronic counselling modules are expected to result in improved interaction and retention of key messages by the participants. Field level android-based devices are web-linked to a central server to leverage the opportunity for real time monitoring of community health workers performance and coverage of intervention delivery. Thus, this study will contribute to the growing body of implementation research on benefits and challenges of integrating electronic platforms in delivering counselling and supplemental interventions in a community setting.

Recently, the government of Bangladesh undertook significant efforts to digitize service delivery in all spheres of health systems including frontline and community level health care providers who are responsible for delivering nutrition interventions [39]. This trial will provide a timely and pertinent contribution to the knowledge base of use of electronic platforms in not only recording and reporting of service utilization but also aiding the community workers in nutrition service delivery at scale.

A limitation of our study includes not being able collect biological samples from study participants due to funding constraints; this would have allowed us to investigate potential biological mediators of the effect of nutrients as well as exploring inherited differences in metabolism predisposing to the risk of stunting. Our study also does not look at knock-on effects beyond 2 years of age.

We anticipate that the results from this cRCT will have policy-level implications in prioritizing a set of interventions when addressing stunting - whether it is behaviour change communication only or combined with prenatal and/or post-natal nutritional supplementation.

## Trial status

Recruitment has been completed. Intervention and data collection is ongoing.

## Additional files

Additional file 1: Composition of LNS for pregnant women. (DOCX 16 kb)

Additional file 2: Composition of LNS for children. (DOCX 17 kb)

## Abbreviations

ANC: Antenatal check-up
BCC: Behaviour change communication
BDHS: Bangladesh Demographic and Health Survey
BMI: Body mass index
CF: Complementary feeding
CFS: Complementary food supplementation
CHW: Community health workers
cRCT: Community-based cluster randomised controlled trial
EBF: Exclusive breastfeeding
FRA: Field research assistant
FW: Field supervisors
HFS: Household food security
IYCF: Infant and young child feeding practices
KAP: Knowledge attitude and practice
LAZ: Length-for-age Z score
LMP: Last menstrual period
LNS: lipid-based nutritional supplement
MIS: Management Information System
MUAC: Mid-upper arm circumference
NGO: Non-government organisations
PNS: Prenatal nutritional supplementation
RDA: Recommended dietary allowance
SD: Standard deviation
SDG: Sustainable development goals
SES: Socio-economic status
WASH: Water, sanitation and hygiene
WHO MGRS Child Growth Reference: WHO Multicentre Growth Reference Study Child Growth Reference
