# Supplementary material for: A community-based cluster randomised controlled trial to evaluate the effectiveness of different bundles of nutrition-specific interventions in improving mean length-for-age z score among children at 24 months of age in rural Bangladesh: study protocol
Source: BMC Public Health. 2017 May 2;17:375. doi: 10.1186/s12889-017-4281-0 (PMC5414300; doi:10.1186/s12889-017-4281-0)
Supplement: Supplementary file 1 — Composition of LNS for pregnant women. (DOCX 16 kb) [file 12889_2017_4281_MOESM2_ESM.docx]

**Additional file 2: Composition of LNS for children**

| **Nutrients** | **For 100g of product** | | **For 20g (recommended daily dose)** |  | **Nutrients** | **For 100g of product** | | **For 20g (recommended daily dose)** |
| --- | --- | --- | --- | --- | --- | --- | --- | --- |
|  | **Min** | **Max** |  |  |  | **Min** | **Max** |  |
| Energy (kcal) | 560 | 620 | 118 |  | Vitamin A (mg) | 1.85 | 2.85 | 0.4 |
| Proteins (g) | 11.7 | 14.3 | 2.6 |  | Vitamin B1 (mg) | 2.5 | 5.5 | 0.5 |
| Lipids (g) | 43.2 | 52.8 | 9.6 |  | Vitamin B2 (mg) | 2.25 | 3.3 | 0.5 |
| Calcium (mg) | 1330 | 1540 | 280 |  | Niacin (mg) | 30 | 38 | 6 |
| Phosphorus (mg) total of which: | 980 | 1130 | 196 |  | Pantothenic acid (mg) | 9.7 | 14.4 | 2 |
| Phosphorus (mg) free | 950 | 1092 | 190 |  | Vitamin B6 (mg) | 2.5 | 3.6 | 0.5 |
| Potassium (mg) | 900 | 1100 | 200 |  | Folic acid (µg) | 750 | 1030 | 150 |
| Magnesium (mg) | 180 | 200 | 40 |  | Vitamin B12 (µg) | 4.4 | 6.5 | 0.9 |
| Zinc (mg) | 36 | 44 | 8 |  | Vitamin C (mg) | 150 | 270 | 30 |
| Copper (mg) | 1.5 | 1.9 | 0.34 |  | Vitamin D (µg) | 24.5 | 44 | 5 |
| Iron (mg) | 40 | 52 | 9 |  | Vitamin E (mg) | 30 | 45 | 6 |
| Iodine (µg) | 383 | 518 | 90 |  | Vitamin K (µg) | 144 | 198 | 30 |
| Selenium (µg) | 80 | 120 | 20 |  |  |  |  |  |
| Manganese (mg) | 4.8 | 7.2 | 1.2 |  |  |  |  |  |
|  |  |  |  |  |  |  |  |  |

Note: 20 g of LNS for children provide a minimum of 4.46g LA (Linoleic Acid) and a minimum of 0.42g ALA (α-Linolenic Acid)
