# Supplementary material for: A community-based cluster randomised controlled trial to evaluate the effectiveness of different bundles of nutrition-specific interventions in improving mean length-for-age z score among children at 24 months of age in rural Bangladesh: study protocol
Source: BMC Public Health. 2017 May 2;17:375. doi: 10.1186/s12889-017-4281-0 (PMC5414300; doi:10.1186/s12889-017-4281-0)
Supplement: Supplementary file 2 — Composition of LNS for children. (DOCX 17 kb) [file 12889_2017_4281_MOESM1_ESM.docx]

**Additional file 1: Composition of LNS for pregnant women**

| **Nutrients** | **For 100g of product** | | **For 20g (recommended daily dose)** |  | **Nutrients** | **For 100g of product** | | | **For 20g (recommended daily dose)** |
| --- | --- | --- | --- | --- | --- | --- | --- | --- | --- |
|  | **Min** | **Max** |  |  |  | **Min** | **Max** |  | |
| Energy (kcal) | 565 | 625 | 119 |  | Vitamin A (µg) | 3720 | 5720 | 800 | |
| Proteins (g) | 11.7 | 14.3 | 2.6 |  | Vitamin B1 (mg) | 14 | 30.8 | 2.8 | |
| Lipids (g) | 46.4 | 56.6 | 10.2 |  | Vitamin B2 (mg) | 12.6 | 18.5 | 2.8 | |
| LA (Linoleic Acid) (g) | 20.1 | 24.5 | 4.46 |  | Niacin (mg) | 180 | 38 | 36 | |
| ALA (α-Linolenic Acid) (g) | 2.1 | 2.6 | 0.47 |  | Pantothenic acid (mg) | 34 | 50.4 | 7 | |
| Calcium (mg) | 1330 | 1540 | 280 |  | Vitamin B6 (mg) | 19 | 27.4 | 3.8 | |
| Phosphorus (mg) | 950 | 1093 | 196 |  | Folic acid (µg) | 2000 | 2750 | 400 | |
| Potassium (mg) | 900 | 1100 | 200 |  | Vitamin B12 (µg) | 25.2 | 37.2 | 5.2 | |
| Magnesium (mg) | 293 | 358 | 65 |  | Vitamin C (mg) | 500 | 900 | 100 | |
| Zinc (mg) | 135 | 165 | 30 |  | Vitamin D3 (µg) | 50 | 88 | 10 | |
| Copper (mg) | 18 | 22 | 4 |  | Vitamin E (mg) | 100 | 150 | 20 | |
| Iron (mg) | 90 | 115 | 20 |  | Vitamin K (µg) | 216 | 297 | 45 | |
| Iodine (µg) | 1063 | 1438 | 250 |  |  |  |  |  | |
| Selenium (µg) | 520 | 780 | 130 |  |  |  |  |  | |
| Manganese (mg) | 10.4 | 15.6 | 2.6 |  |  |  |  |  | |
|  |  |  |  |  |  |  |  |  | |
